# Comparative Assessment of the Bioremedial Potentials of Potato Resistant Starch-Based Microencapsulated and Non-encapsulated *Lactobacillus plantarum* to Alleviate the Effects of Chronic Lead Toxicity

**DOI:** 10.3389/fmicb.2018.01306

**Published:** 2018-06-19

**Authors:** Zafarullah Muhammad, Rabia Ramzan, Shanshan Zhang, Haijuan Hu, Ahsan Hameed, Amr M. Bakry, Yongzhen Dong, Lufeng Wang, Siyi Pan

**Affiliations:** ^1^Key Laboratory of Environment Correlative Dietology, Ministry of Education, Huazhong Agricultural University, Wuhan, China; ^2^Food Biotechnology and Food Safety Laboratory, Huazhong Agricultural University, Wuhan, China; ^3^School of Agricultural Engineering and Food Science, Shandong University of Technology, Zibo, China

**Keywords:** bioremediation, potato resistant starch, *L. plantarum*, microencapsulation, chronic lead toxicity, oxidative stress

## Abstract

Lead (Pb) is a well-recognized and potent heavy metal with non-biodegradable nature and can induce the oxidative stress, degenerative damages in tissues, and neural disorders. Certain lactic acid bacterial strains retain the potential to mitigate the lethal effects of Pb. The present work was carried out to assess the Pb bio-sorption and tolerance capabilities of *Lactobacillus plantarum* spp. Furthermore, potato resistant starch (PRS)-based microencapsulated and non-encapsulated *L. plantarum* KLDS 1.0344 was utilized for bioremediation against induced chronic Pb toxicity in mice. The experimental mice were divided into two main groups (Pb exposed and non-Pb exposed) and, each group was subsequently divided into three sub groups. The Pb exposed group was exposed to 100 mg/L Pb(NO_3_)_2_ via drinking water, and non-Pb exposed group was supplied with plain drinking water during 7 weeks prolonged *in vivo* study. The accumulation of Pb in blood, feces, renal, and hepatic tissues and its pathological damages were analyzed. The effect of Pb toxicity on the antioxidant enzyme capabilities in blood, serum, as well as, on levels of essential elements in tissues was also calculated. Moreover, KLDS 1.0344 displayed remarkable Pb binding capacity 72.34% and Pb tolerance (680 mg/L). Oral administration of both non- and PRS- encapsulated KLDS 1.0344 significantly provided protection against induced chronic Pb toxicity by increasing fecal Pb levels (445.65 ± 22.28 μg/g) and decreasing Pb in the blood up to 137.63 ± 2.43 μg/L, respectively. KLDS 1.0344 microencapsulated with PRS also relieved the renal and hepatic pathological damages and improved the antioxidant index by inhibiting changes in concentrations of glutathione peroxidase, glutathione, superoxide dismutase, malondialdehyde, and activated oxygen species, which were affected by the Pb exposure. Overall, our results suggested that *L. plantarum* KLDS 1.0344 either in free or encapsulated forms hold the potentiality to deliver a dietetic stratagem against Pb lethality.

## Introduction

A prompt industrial expansion, farm mechanization, and indiscreet utilization of agricultural manures, invigilancy to dispose-off solid wastes, and inappropriate mining operations are imperiling the human health and ecosystems because of the constant discharge of lethal heavy metals (Huang et al., 2015; Lai et al., 2016; Sidhu et al., 2016; Zhang C. et al., 2016; Liang et al., 2017a). Lead (Pb) has been documented as a critical, heavy metal contaminant, and it possesses grievous eco-toxicological indications with the elongated persistency in the environment due to its non-biodegradable behavior (Flora et al., 2012; Liang et al., 2017b). Even at very low concentrations, Pb is incredibly pestilent, and it is generally accumulated in the kidneys, liver, bones (Patra et al., 2001), and muscles instigating numerous dreadful ailments, like oxidative stress (Patra et al., 2001; Khordad et al., 2013), carcinogenesis (Valverde et al., 2002), disturbance of calcium homeostasis, degenerative variations (Sansar et al., 2011, 2012), and tissue illnesses, mostly in the kids (Ahyayauch et al., 2013).

Numerous researches suggest that oxidative stress is among the mechanisms significantly involved in the baneful effects of Pb (Hosseini et al., 2015). Chelation therapy is the most typical treatment for heavy metal lethality by utilizing the chelating agents such as Meso-2,3-dimercaptosuccinic acid (DMSA) and calcium disodium versenate (CaNa_2_EDTA). However, adversative effects, including skin reactions, malaise, renal injury, vomiting, and nausea occur (Bayer, 2015). These chelating agents can also cause subsequent deficiencies of zinc, copper, and other crucially important trace nutrients, which are a vital part of the body’s antioxidant defense system (Yadav et al., 2014). According to several studies, kidney is the main organ in the body where the development of chronic Pb toxicity can occur (Bidlack, 2002; Brzóska et al., 2003). Howsoever, most part of the absorbed Pb all through chronic exposure is originally deposited in the liver before its transportation to the kidney by the bloodstream (Zhai et al., 2014; Javed et al., 2016; Morcillo et al., 2016; Zhang S. et al., 2016; Ashraf and Tang, 2017).

As sorption matrices, there are several biodegradable natural polymers that have increased attention because of their metal chelating competence, simplicity of processing, non-toxicity, and worldwide afford-ability (Li and Buschle-Diller, 2017). Recently, some plant polymer extracts have been found to have the capacity to decrease tissue Pb levels and Pb-induced oxidative stress (Yadav et al., 2014; Liu et al., 2018). Owing to low cost, biodegradability and renewability starch is considered among the agricultural biopolymers that are expansively utilized in the food and food-related industry (Soto et al., 2015, 2016; Peng et al., 2016). The potato resistant starch (PRS) is consisted of compact structured granules of native potato starch, having resistance to enzymatic hydrolysis and higher contents of amylopectin (Dragan et al., 2014; Cieśla et al., 2015). Potato starch possesses unique features to form Werner-type complexes (Ciesielski and Tomasik, 2004a,b), and these complexes contain the ability to ligate a metal atom to the lone electron pairs of hydroxyl groups from phosphate groups and D-glucose units in the starch (Bhat et al., 2015; Soto et al., 2016). The potentially possible sites for metal ions interaction with polysaccharide chains are the hydroxyl groups of D-glucose units of starch granules and in this regard, the PRS has greater ability to bind cations (Blennow et al., 2000, 2006; Ciesielski and Tomasik, 2004a). Bhat et al. (2015) conducted a study to assess the efficiency of PRS against heavy metals and stated that it could be effective adsorbent with 78.1% efficiency against Pb adsorption.

According to some studies, anti-oxidative defense system in lactic acid bacteria (LAB) either enzymatic or non-enzymatic might critically perform its role in detoxifying the damages produced by the oxidative stress (Halttunen et al., 2008; Huang et al., 2017; Yi et al., 2017). Furthermore, LAB are recognized to possess anti-oxidative characteristics in human, which might be additional imperative property to provide protection against Pb-induced noxiousness (Monachese et al., 2012). Bhakta et al. (2012), Tian et al. (2012), and Zhai et al. (2013, 2015), conducted studies on the healing effects of oral administration of the LABs’ and their shielding effect against Pb noxiousness by reducing Pb levels in the tissues and blood and, impeding Pb-induced oxidative strain. The study conducted by Li et al. (2017) has also provided the information that some *Lactobacillus* strains possess a positive impact to alleviate the acute Pb toxicity. In our previously published work (Muhammad et al., 2017), we successfully incorporated PRS for the microencapsulation of *Lactobacillus Plantarum* KLDS 1.0344.

Presently, there is no specific natural remedy has been developed to deal with the chronic Pb poisoning. Therefore, introducing some new strategies tackling chronic Pb toxicity is the dire need of the day. To the best available information, the healing effects of *L. plantarum* 1.0344 microencapsulated with polysaccharides, like PRS, have not been studied so far to remedify the chronic Pb toxicities. Based on the findings of our previous (Muhammad et al., 2017) studies, the present work has been designed to appraise the alleviation and adsorption of Pb by PRS-microencapsulated and non-encapsulated *L. plantarum* KLDS 1.0344 and, their effects on anti-oxidative responses (enzymatic and non-enzymatic), oxidative stress, as well as, the Pb tolerance and anti-oxidative mechanisms, decrease in intestinal Pb absorption, reduction of Pb accretion in tissues, and alleviation of hepatic and renal oxidative stress have been assessed. Overall, the aims of the current study were set to evaluate and to compare the role of *L. plantarum* 1.0344 in non-encapsulated and microencapsulated forms for the alleviation of the induced chronic Pb toxicity by using the adult female BALB/c mice as a model animal.

## Materials and Methods

### Chemicals and Reagents

BH 2100 kit (Beijing Bohui Innovation Technology Co., Ltd) was used to determine the levels of Pb from the whole blood samples. The kits Njjcbio A003, Njjcbio A006, Njjcbio A005, Njjcbio A001, Njjcbio A007, Njjcbio C010, and Njjcbio C009 were used to determine the levels of malondialdehyde (MDH), glutathione (GSH), GSH peroxidase (GSH-Px), superoxide dismutase (SOD), catalase (CAT), aspartate aminotransferase (AST), and alanine aminotransferase (ALT), respectively. These kits were procured from the Jiancheng Bioengineering Institute (Nanjing, China). PRS (CAS: 9005-25-8) was purchased from the Aladdin Industrial Corporation, Shanghai, China. Pb nitrate and other analytical laboratory chemicals and reagents were purchased from the Sinopharm Chemical Reagent Company (Shanghai, China), δ-aminolevulinic acid was procured from Chuanxiang Bioengineering Company (Wuhan, China).

### Bacterial Strain and Culture

*Lactobacillus plantarum* KLDS strains were isolated from the traditional fermented cheese in Inner Mongolia and were stored in KLDS (Key Laboratory of Dairy Science) under the patronage of Chinese Ministry of Education. Frozen stock culture of *L. plantarum* strains were reactivated twice in MRS (de Man, Rogosa and Sharpe) broth, and incubation was done at 37°C up to the stationary phase. Then, culture was centrifuged at 4°C for 10 min at 10,000 *g* to collect the cell pellets. Thermo Sorvall Legend Micro, 21 microcentrifuge was used for the centrifugation purpose. The pellets were again washed three times and suspended in distilled and sterilized water.

### Microencapsulation, Survivability During Storage, and *in Vitro* Digestion

The probiotics (*L. plantarum* KLDS 1.0344) were microencapsulated by using PRS. Buchi B-290, Flawil, Switzerland (laboratory-scale spray dryer) was used to execute the microencapsulation process. Survivability during storage and *in vitro* digestion was carried out as formerly reported in our previously published article (Muhammad et al., 2017). As the formulations with PRS gave us the best results during microencapsulation process, storage, and *in vitro* digestibility in the previous study, we selected these microencapsulated strains for the animal experiments. Lyophilization of the microencapsulated cells was done by mixing the skim milk with the encapsulated cells, and cells were then stored at -20°C. Skim milk was utilized as a cryoprotectant agent. Before doing the animal experiments, viability of the lyophilized cells was done by the colony counting method. In short, the freeze-dried cells were reactivated with the ultrapure water. After the colony counting, viability of the cells was documented as around 2.7 × 10^9^ CFU/mL. Thereof, the mice were orally gavage 0.5 mL of the reactivated lyophilized cells in skimmed milk, and it is conformed to 1.3 × 10^9^ CFU of the bacterial cells.

### Appraisal of the Binding and Tolerance of Pb

The ability of seven KLDS strains to bind the Pb was investigated as formerly described by Li et al. (2017), with little amendments. Incubation of all strains was carried out for 16 h, and the centrifugation of the cultivated biomass was done at 10,000 g for 10 min at 4°C. The cell pellets were obtained after two times washing of the centrifuged mass with ultrapure water. Concentration of the bacteria was set to 1 g/L (wet weight) with ultrapure water having 100 mg/L lead nitrate, and incubation of the samples was done for 24 h at 37°C. Afterwards, centrifugation of the samples was carried out for 20 min at 10,000 g, and the concentrations of the residual Pb in the supernatants were determined by (Spectra AA 220; Varian, Palo Alto, CA, United States) flame atomic absorption spectrophotometer. Following equation was used to determine the metal removal efficiency on the basis of mass balance in Equation (1).ˆ

$$\text{Removal}(\%)=\frac{C_{i}-C_{e}}{C_{i}}\times 100$$

where *C*_e_ and *C*_i_ are the respective residual Pb concentrations and initial Pb concentrations after removal. The MIC (Minimum inhibitory concentration) approach was followed to determine the Pb tolerance of each strain according to the methods as described by Abou-Shanab et al. (2007) and Gupta et al. (2012). Preparation of the MRS agar medium having (100 to 1000 mg/L) lead nitrate solution was carried out. Each cultured LAB strain (10 μL) was spread on the MRS agar medium and the inoculum level was taken as 1 × 10^9^ CFU/mL. Then incubation was done at 37°C for 48 h, and subsequently the LAB growth was determined. In the present study, the least concentration of Pb that entirely repressed the LAB growth was taken as MIC.

### Comparison of Protective Potentials of Non-encapsulated and Microencapsulated *L. plantarum* KLDS 1.0344 Against Chronic Pb Toxicity *in Vivo* Study

#### Experimental Design of the Animal Trial

The mice used in all of the animal experiments were adult female BALB/c mice, and these were procured from the Beijing Vital River Lab. Animal Technology Co., Ltd. (China). Stainless steel cages and a well-equipped room to retain a 12 h light-dark cycle, controlled humidity, and temperature were used to keep the mice. Mice were given the standard commercial mouse food, and Pb-containing and Pb-free water were also supplied *ad libitum* in therapy groups. The entire protocols for the present study were approved by the Ethics Committee of Huazhong Agricultural University and Hubei provincial Animal Care Committee, Wuhan, China. The procedures and techniques of this study were strictly followed according to the European Community guidelines for the use and care of experimental animals (directive 2010/63/EU).

Such as presented in **Table 1**, the mice were distributed into six subgroups, control (no Pb or KLDS 1.0344), Pb only, non-encapsulated *L. plantarum* KLDS 1.0344 only, encapsulated *L. plantarum* KLDS 1.0344 only, Pb plus non-encapsulated *L. plantarum* KLDS 1.0344, and Pb plus encapsulated *L. plantarum* KLDS 1.0344 with 10 mice in every respective group. The mice were given orally a dosage of 1.3 × 10^9^ CFU of *L. plantarum* KLDS 1.0344 mixed in 0.5 mL of skim milk through gavage once in a day. Pb exposure of the mice was done by (Pb containing) drinking water, 100 mg/liter (0.05 mg/kg-day)^-1^ oral dose of Pb from lead nitrate in drinking water was taken for modeling of environment related concentration of Pb exposure) (Office of Environmental Health Hazard Assessment [OEHHA], 2001; Al-Khfaji et al., 2011). Measurements of the body weight were done during the treatment period of 7 weeks. The fecal samples were collected at the end of every week while, each mouse was relocated into an empty and clean cage for 1 h. Mice were individually moved to metabolic cages and kept there for 24 h at the end of the 7th week. Latterly, under light ether anesthesia, all the mice were sacrificed, and the blood was taken in heparinized tubes in order to obtain plasma.

**Table 1:** Protocol for the animal experiment.

	Groups	Feeding plan
Non-lead exposed	Control	SM+PW
	Non-encapsulated *L. plantarum* KLDS 1.0344	SM+PW+KLDS 1.0344
	Encapsulated *L. plantarum* KLDS 1.0344	SM+PW+encapsulated KLDS 1.0344
Lead exposed	Lead only	SM+Pb (in drinking water)
	Non-encapsulated *L. plantarum* KLDS 1.0344+lead	SM+Pb (in drinking water)+KLDS 1.0344
	Encapsulated *L. plantarum* KLDS 1.0344+lead	SM+ Pb (in drinking water)+encapsulated KLDS 1.0344

*Skimmed milk (SM) daily 0.5 mL through gavage; Plain drinking water (PW); Pb (in drinking water), Lead nitrate at 100 mg/L (in drinking water); SM+non-encapsulated and PRS-encapsulated KLDS 1.0344, 1.3 × 10^9^ CFU L. plantarum KLDS 1.0344 in skimmed milk (SM) daily 0.5 mL through gavage. The experimental treatments lasted for 7 weeks.*

After scarifying the animals, the kidneys and livers were removed, and saline solution (prepared with sodium chloride and sterilized distilled water) was utilized to wash the samples. Prior to histopathological studies, the samples were removed and preserved in formalin saline (10%) for 48 h. The remaining kidney and liver samples were wrapped in aluminum foils, put into the Eppendorf tubes and stored at -80°C to assess the chemical elements and biochemical assays (Zhai et al., 2014, 2015).

#### Quantifying Chemical Elements in Tissues

The tissue samples were moved to digestion vessels. The vessels were metal free, manufactured by Omni; CEM, United Kingdom. Then, samples were digested in concentrated nitric acid by using the Microwave Digestion System manufactured by MARS; CEM, United Kingdom. Concentrations of calcium (Ca), zinc (Zn), magnesium (Mg), and iron (Fe) in the kidneys and liver were measured with the use of graphite furnace or flame atomic absorption spectrophotometer (Zhai et al., 2014).

#### Measurement of Pb in the Blood, Feces, and Tissues

BH 2100 kit (Beijing Bohui Innovation Technology Co., Ltd.) was used to determine the levels of Pb from the whole blood samples. The samples of feces and tissues were digested in a microwave digestion system using concentrated HNO_3_. The concentration of Pb in the feces, kidneys, and livers was measured by an atomic absorption spectrophotometer, i.e., graphite furnace or flame spectrophotometer (Specter. AA; Varian or AAS). The quantity of Pb was given in mg/g of feces (wet weight) and μg/g of tissue (Dobrakowski et al., 2016).

#### Biochemical Assays

The MDH, GSH level, activities of GSH-Px, SOD, CAT in the mice tissues while, AST and ALT in the serum were determined with the respective kits obtained from the Jiancheng Bioengineering Institute. All assays were done in triplicate in accordant to the manufacturer’s guidelines (Dobrakowski et al., 2016).

#### Histopathological Analyses

The kidney and liver tissues were split into 5 μm thickness with rotary microtome and fixed in paraffin. These sliced tissues were stained with hematoxylin-eosin and then observed by light microscopy (Li et al., 2017).

### Statistical Analyses

Entire analyses were completed in triplicate, and the data were compiled. Tukey method was utilized by using the statistical program Statistics 8.1 (Analytical Software, United States) for analysis of variance (ANOVA) and comparison of means. Statistically, the difference was considered as significant when the *P* < 0.05. Standard errors and mean values were deliberated and demonstrated in charts as coordinate pairs with error bars.

## Results

### Lead Biosorption and Tolerance of the LAB Strains

**FIGURE 1:**
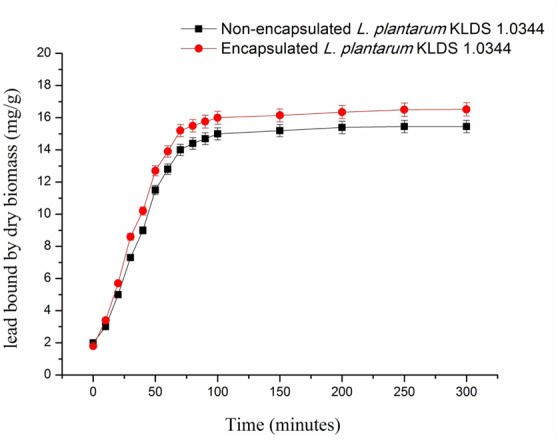
Lead of binding of Non-encapsulated and PRS-based encapsulated *L. plantarum* 1.0344 at different time points. Values are given as mean ± standard deviation (SD).

Abilities of seven *Lactobacillus* KLDS strains to bind and tolerate the Pb have been presented in **Table 2**. The tested strains showed a range of 20.23 ± 0.51% to 72.34 ± 2.55% to remove the Pb (**Table 2**), while initially the concentration of Pb was 100 mg/L. Among all the seven tested strains *L. plantarum*, KLDS 1.0344 exhibited the greatest binding ability. On the basis of the binding ability *L. plantarum*, KLDS 1.0344 was taken as the best suitable candidate to be investigated for further *in vivo* assays. Additionally, PRS-encapsulated *L. plantarum* KLDS 1.0344 also showed more Pb binding ability followed by the non-encapsulated bacteria (**Figure 1**).

**Table 2:** The Pb-binding ability and Pb tolerance of the tested *Lactobacillus plantarum* KLDS (*L. plantarum* KLDS) strains.

Strains	Lead removal by wet biomass (%)	Minimum inhibitory concentration for Pb (mg/L)
*L. plantarum* KLDS 1.0317	36.12 ± 1.27ˆf	450.00 ± 7.09ˆb
*L. plantarum* KLDS 1.0318	61.71 ± 1.14ˆb	200.00 ± 6.89ˆd
*L. plantarum* KLDS 1.0344	72.34 ± 2.55ˆa	680.00 ± 4.04ˆa
*L. plantarum* KLDS 1.0386	51.22 ± 1.81ˆd	100.00 ± 3.53ˆe
*L. plantarum* KLDS 1.0628	43.86 ± 1.55ˆg	150.00 ± 5.32ˆf
*L. plantarum* KLDS 1.0985	58.09 ± 0.73ˆc	350.00 ± 2.98ˆc
*L. plantarum* KLDS 1.0986	20.23 ± 0.51ˆg	50.00 ± 1.76ˆg

*Incubation was done with an initial lead nitrate concentration of 100 mg/L. Values are mean ± SD of three determinations. Different letters indicate the significant differences (P < 0.05) among the strains.*

### Body Weight of Mice

**FIGURE 2:**
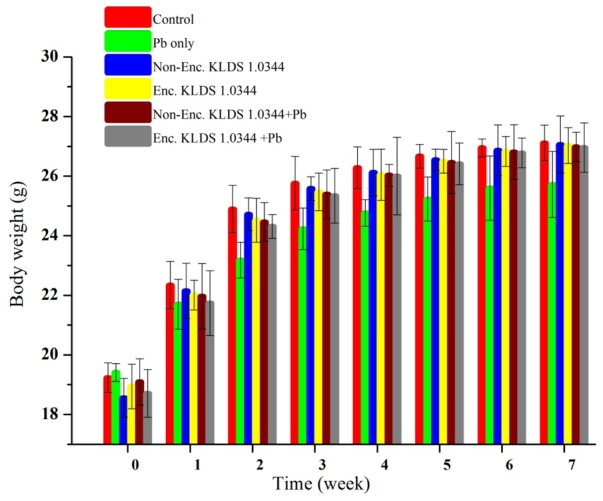
Evaluating the effect of Non-encapsulated and PRS-based encapsulated *L. plantarum* 1.0344 on body weights of the mice with lead provided in drinking water, throughout the experimental period of 7 weeks. Values are given as mean ± standard deviation (SD).

On the basis of Pb biosorption and tolerance of the LAB strains, we selected *L. plantarum* KLDS 1.0344 to evaluate its effect on the Pb toxicity in the forms of microencapsulated and non-encapsulated bacteria. Increment of weight was observed in every mouse through 7 week experimentation, and there was no significant (*P* > 0.05) difference was found among all the groups. Maximum body weight witnessed in control 27.11 ± 0.6 g succeeding by 26.96 ± 0.83 g in PRS microencapsulated KLDS 1.0344+Pb group, contrary to the Pb group only (25.72 ± 1.11 g) at 7th week given in **Figure 2**.

### Pb Contents in the Excreta, Liver, Kidneys, and Blood

**FIGURE 3:**
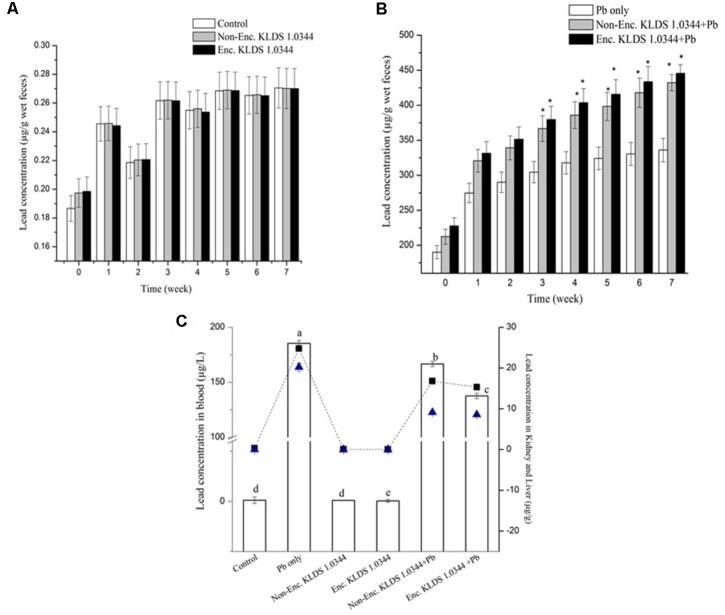
Impact of feeding the Non-encapsulated and PRS-based encapsulated *L. plantarum* 1.0344 on the levels of Pb in the feces of mice throughout the experimental period of 7-weeks: **(A)** Levels of Pb in the feces of mice without lead nitrate in drinking water; **(B)** levels of Pb in the feces of mice with lead nitrate in drinking water. The significant difference (*P* < 0.05) shown by ^∗^; and **(C)** levels of Pb in the blood (μg/L) of mice presented with bar, in kidney (μg/g) of mice presented with 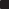 with dotted line, in liver (μg/g) of mice presented with 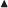 of mice with lead nitrate in drinking water. The significant difference (*P* < 0.05) shown by letters. Values are given as mean ± standard deviation (SD).

The Pb concentrations in excreta of mice (groups) given Pb orally, were suggestively greater than all of the other (deprived of Pb in drinking water) groups (*P* < 0.01). The variations in the Pb concentrations in excreta are demonstrated in the Pb oral (**Figure 3B**) and Pb free oral (**Figure 3A**) groups. In Pb free oral groups, the lower most Pb intensity in excreta was perceived in control 0.18 ± 0.00 μg/g (wet) feces during first week (**Figure 3A**). While, in Pb oral groups, the orally given PRS encapsulated KLDS 1.0344+Pb were substantially (*P* < 0.05) incremented fecal Pb quantities, compared to the Pb-only group, after each week of the experimentation. The highest Pb fecal concentrations (445.6 ± 12.2 μg/g wet feces) were displayed by the PRS encapsulated KLDS 1.0344+Pb only oral groups followed by the KLDS 1.0344+Pb in 7th week (**Figure 3B**). During the 7 week study, the elimination quantities of Pb in the Pb group and therapy groups (including the KLDS 1.0344 in skim milk given through gavage) were smaller in all successive weeks, indicating that PRS encapsulated and non-encapsulated KLDS 1.0344 were potentially effective against the chronic Pb toxicity.

The concentrations of Pb in the kidneys, liver, and blood in the non-orally exposed Pb groups were significantly smaller than the orally exposed Pb groups (*P* < 0.05) of entire cliques and are publicized in **Figure 3C**. In the kidneys and blood, the declining levels of Pb in all Pb dose groups were suggestively different from the results noticed for the Pb group only (*P* < 0.05). Likened with the Pb-only groups, non-encapsulated and PRS encapsulated *L. plantarum* KLDS 1.0344 treatments expressively decreased the Pb contents in the blood and tissues. The tissue Pb contents in the control, non-encapsulated, and PRS encapsulated KLDS 1.0344-only groups were very low in the liver and 0.30 μg/g ± 0.04, 0.05 ± 0.008 μg/g, 0.03 ± 0.001 μg/g, respectively, in the kidney in **Figure 3C**. Comparing with the orally exposed Pb only (20.22 ± 0.98 μg/g in liver 24.8 ± 0.6 μg/g in kidney), non-encapsulated KLDS 1.0344+Pb (9.11 ± 0.06 μg/g in liver 16.78 ± 0.5 μg/g in kidney), and PRS encapsulated KLDS 1.0344+Pb (8.56 ± 0.09 μg/g in liver 15.32 ± 0.7 μg/g in kidney) significantly (*P* < 0.05) decreased the concentrations of Pb in the hepatic and renal tissues of the mice. The Pb contents in blood (μg/L) of mice are presented in **Figure 3C**. As compared to the Pb-only group (185.46 ± 2.57 μg/L), PRS encapsulated *L. plantarum* KLDS 1.0344+Pb (137.63 ± 2.44 μg/L) considerably reduced the Pb concentration in blood and tissues and provided protection to counter Pb burden in the blood and tissues.

### Ca, Zn, Fe, and Mg Quantities in the Kidneys and Liver

Exposure of the Pb brought about variations of the Zn, Ca, Mg, and Fe metal quantities in both the liver and the kidneys (**Tables 3A,B**, respectively). Maximum of these changes were suggestively inverted in the non-encapsulated and encapsulated KLDS 1.0344+Pb groups (*P* < 0.05) in kidneys except for zinc. However, in liver, significant results were witnessed for increased metal levels in all others groups as compared to the Pb group only.

**Table 3:** **(A)** Effects of non-encapsulated and PRS-based encapsulated *L*. *plantarum* 1.0344 on metal levels in the livers of mice. **(B)** Effects of non-encapsulated and PRS-based encapsulated *L*. *plantarum* 1.0344 on metal levels in the kidneys of mice.

Group	Zn	Ca	Mg	Fe
**(A)**				
Control	26.35 ± 1.32	288.45 ± 1.42ˆd	257.81 ± 4.89ˆa	80.23 ± 1.01ˆa
Lead group	27.68 ± 1.40	225.90 ± 2.30ˆe	232.33 ± 3.62ˆd	40.65 ± 2.03ˆe
Non-encapsulated *L. plantarum* KLDS 1.0344	25.57 ± 1.28	300.28 ± 3.01ˆbc	240.46 ± 6.02ˆbc	76.65 ± 3.83ˆb
Encapsulated *L. plantarum* KLDS 1.0344	25.96 ± 1.30	322.45 ± 7.12ˆa	244.18 ± 5.21ˆb	79.11 ± 3.96ˆab
Non-encapsulated *L. plantarum* KLDS 1.0344 + lead	27.49 ± 1.70	294.67 ± 3.73ˆc	236.74 ± 1.84ˆcd	54.69 ± 0.73ˆd
Encapsulated *L. plantarum* KLDS 1.0344 +lead	27.24 ± 1.62	307.19 ± 5.36ˆb	239.52 ± 3.98ˆc	58.22 ± 2.91ˆc
**(B)**				
Control	17.22 ± 0.86	800.13 ± 7.01ˆbc	190.34 ± 3.52ˆe	55.26 ± 2.76ˆd
Lead group	20.15 ± 1.01	807.75 ± 1.39ˆb	218.90 ± 7.95ˆbc	50.59 ± 2.53ˆe
Non-encapsulated *L. plantarum* KLDS 1.0344	19.26 ± 0.96	794.32 ± 2.72ˆd	224.56 ± 0.23ˆb	62.41 ± 0.12ˆb
Encapsulated *L. plantarum* KLDS 1.0344	19.34 ± 0.97	799.68 ± 3.98ˆc	238.12 ± 1.91ˆa	64.25 ± 1.21ˆa
Non-encapsulated *L. plantarum* KLDS 1.0344 + lead	19.67 ± 0.88	812.89 ± 4.64ˆa	203.27 ± 9.16ˆd	58.03 ± 2.90ˆcd
Encapsulated *L. plantarum* KLDS 1.0344 +lead	19.83 ± 0.99	817.26 ± 5.86ˆa	217.62 ± 3.80ˆc	59.78 ± 1.99ˆc

*Data are expressed as mean ± standard deviation. According to Tukey means post comparison test, different letters within different rows designate the significant difference.*

### MDA, GSH, SOD, GPx, and CAT in Liver and Kidneys

**FIGURE 4:**
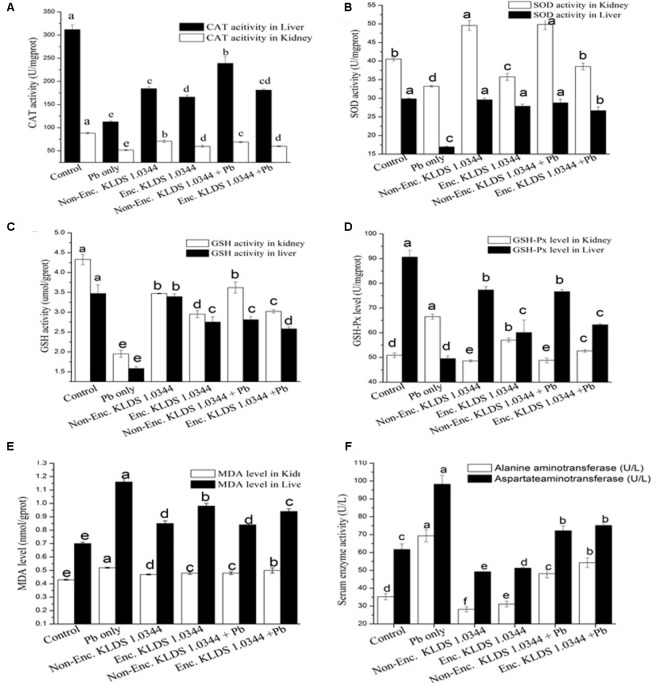
Effects of Non-encapsulated and PRS-based encapsulated *L. plantarum* 1.0344 on Pb-induced oxidative stress, differences of antioxidant capability in the kidney and liver as well as activity of marker enzymes in the serum of mice: **(A)** catalase (CAT); **(B)** superoxide dismutase (SOD); **(C)** glutathione (GSH); **(D)** glutathione peroxidase (GSH-PX); **(E)** malondialdehyde (MDA); and **(F)** aspartate aminotransferase (AST) and alanine aminotransferase (ALT). Values are given as mean ± SD.

The antioxidant capacity levels in the kidney and liver of mice are presented in **Figure 4**. In both orally and non-orally exposed Pb groups, chronic Pb exposure initiated noticeable reduction in levels of CAT (**Figure 4A**), GSH (**Figure 4C**), and SOD (**Figure 4B**), accompanied by increased concentrations of MDA (**Figure 4E**) and GSH-Px (**Figure 4D**). Utilization of the PRS encapsulated *L. plantarum* 1.0344 treatment was efficacious in maximizing CAT (181.21 ± 2.16 U/mgprot as compared to the Pb only group 112.62 ± 2.03 U/mgprot in liver), GSH and SOD degrees and decreasing MDA and GSH-Px (52.64 ± 0.54 U/mgprot as compared to the Pb only group 66.48 ± 1.09 U/mgprot in liver) levels (*P* < 0.05). KLDS 1.0344 either in non-encapsulated or PRS-based encapsulated form had substantial protecting properties on the antioxidant capacity (*P* < 0.05). Co-treatment with non-encapsulated and PRS encapsulated KLDS 1.0344+Pb significantly protected against the variations of these parameters in the oral introduction. In kidneys prolonged Pb acquaintance produced an apparent increase in MDA and GSH-Px, whereas, there was improvement in CAT, GSH, and SOD activity. The activity of the marker enzymes ALT and AST in the serum of mice are given in **Figure 4F**.

### Histopathological Appraisal

**FIGURE 5:**
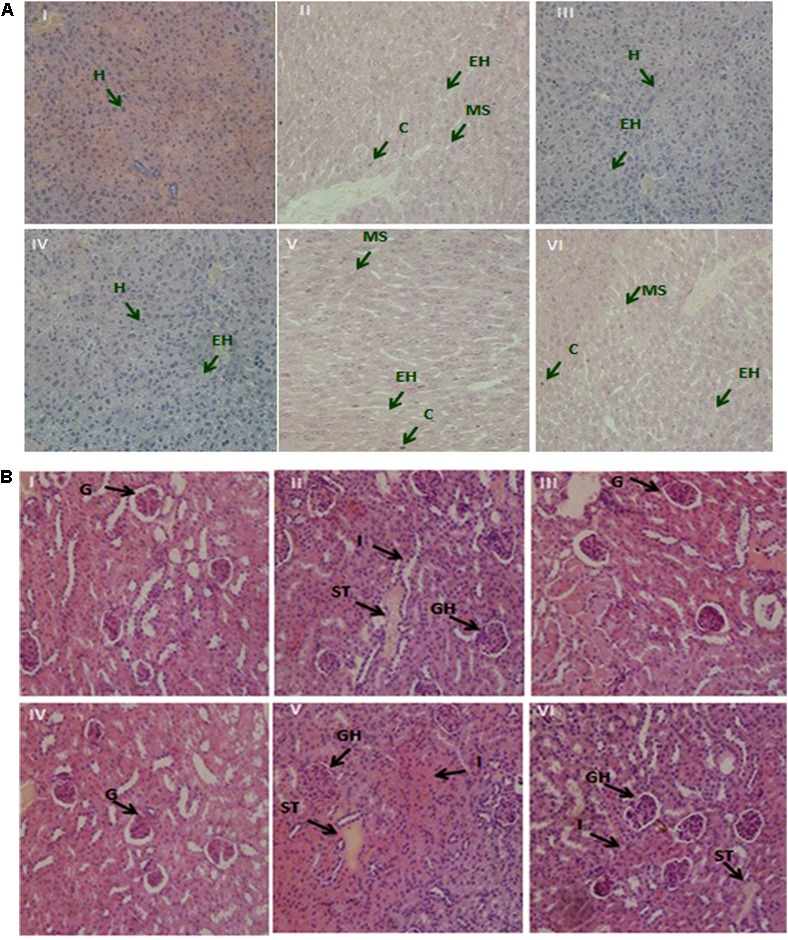
**(A)** The demonstrative micrographs of the hepatic (liver) tissues of the mice with hematoxylin-eosin (H&E staining; magnifications × 400). (I) Control, (II) Lead only, (III) Non-encapsulated *L. plantarum* KLDS 1.0344 only, (IV) PRS encapsulated *L. plantarum* KLDS 1.0344 only, (V) Non-encapsulated *L. plantarum* KLDS 1.0344 plus lead, (VI) PRS encapsulated *L. plantarum* KLDS 1.0344 plus lead. Exposure to lead produced histological alterations in the liver, comprising chromatin condensation (C), injury of intact liver plates and cytoplasmic vacuolization. While in case of liver histopathological examination the lead only group (II), showed vacuolar degeneration, enlarged hepatocytes (EH) and microvesicular steatosis (MS). No obvious histopathological changes were noticed in control (I). While with the exemption of certain hepatocytes (H) swelled observed co-treatment with Non-encapsulated and PRS encapsulated KLDS 1.0344 with lead indicated protecting properties against identical liver injuries (V, VI). **(B)** Demonstrative micrographs of renal (kidney) tissues of the mice with hematoxylin-eosin (H&E staining; magnifications × 400). (I) Control, (II) Lead only, (III) Non-encapsulated *L. plantarum* KLDS 1.0344 only, (IV) PRS encapsulated *L. plantarum* KLDS 1.0344 only, (V) Non-encapsulated *L. plantarum* KLDS 1.0344 plus lead, (VI) PRS encapsulated *L. plantarum* KLDS 1.0344 plus lead. There was normal histomorphology of the kidney was obviously apparent in the control groups (I). Treatments with Non-encapsulated and PRS-based encapsulated KLDS 1.0344 with lead considerably relieved such renal damages. While in lead only group (II) the volume of the glomeruli (G) turned significantly bigger i.e. glomeruli were hyperemic, while some of the glomeruli were found missing, swelling was also observed in some renal tubular epithelial cells (II, IV,VI). Symptoms like glomerular hyperemic (GH), granular degeneration, swollen tubular (ST) epithelial cells and inflammatory cells (I) of all treated groups (III, IV, V, VI) were improved as compared to the lead (Pb) group.

Prolonged Pb contact instigated a notable damage to the mice tissues. Demonstrated photomicrographs of liver and kidney tissue specimen from all groups are presented in **Figures 5A,B**. Treatments with Pb plus non-encapsulated and PRS encapsulated KLDS 1.0344 considerably relieved such hepatic damages. In the kidneys, cloudy swelling and necrosis of tubules and enlargement of glomeruli were apparent in Pb only treated animals, contrary to the control group. It was witnessed likewise that, the renal and hepatic morphology of the non-encapsulated and PRS encapsulated KLDS 1.0344 groups was not varying from that of the control group.

## Discussions

In the emerging nations like China, India, and Thailand, Pb noxiousness has been the utmost premeditated communal health evils, particularly for specific hazardous professions including painting and mining (Ibrahim et al., 2006; Halttunen et al., 2007) The use of biomass such as bacteria, fungi, and algae as bio-sorbents have great potentials for cleaning up the environmental contaminants due to the availability of a broad range of sorption sites which can, not only, retain metallic ions and metals but also several other organic materials (Jiménez-Pranteda et al., 2012; Kinoshita et al., 2013). The chelation treatment has been found deficient of safety and effectiveness, leading us to strive for certain novel approaches against Pb lethality. *Lactobacilli* are found to have capabilities to bind and eliminate numerous heavy metals like Pb, cadmium, and copper *in vitro* (Ibrahim et al., 2006; Halttunen et al., 2007; Schut et al., 2011). A variety of approaches are employed in bioremediation and among these approaches, encapsulation of bacterial cells is considered to be the most promising due to their potential in removing specific contaminants within a carrier matrix (Bruschi et al., 2003). Encapsulation has variety of auspicious implications in multiple fields, from bioreactors to numerous medicines. Protection from severe external environments, easiness of separation, and a decreased vulnerability to adulteration by external organisms are the potential benefits of encapsulated cells. Coacervation and/or emulsifying-crosslinking and spray-drying are among the most common encapsulation approaches (San Keskin et al., 2018). There are several encapsulating compounds that have been utilized for these objectives. A frequent use of polysaccharides for microencapsulation is due to the reason as they have constituents or matrices which could be degraded by intestinal microorganisms and, consequently, allow and support the probiotics to be delivered at targeted sites in the human intestine (de Vos et al., 2010; Mahmoud et al., 2015).

Lead is a widespread pollutant present into the environment with a negative impact on biological activities. It causes adverse damage to the host by inducing oxidative stress. According to several researchers LAB have been reported to possess the ability to give relief from oxidative stress by sequestering the Pb and repairing the damage caused by Pb induced oxidative stress (Fewtrell et al., 2004; Okoro et al., 2015). Numerous properties should be taken into considerations while screening out the LAB against Pb noxiousness. First of all, the LAB should possess the higher capability to bind the Pb, facilitating them to attach the Pb before the Pb could be absorbed into the intestine of the host. The ability to resist the Pb to avoid Pb poisonous is the second pre-requisite of the LAB strains. Furthermore, it is obligatory for the screened strains to retain viability into the highly acidic concentrations and harsh environments of bile and stomach to accomplish Pb elimination in the gastrointestinal tract. In our present investigation, among the seven tested strains the *L. plantarum* KLDS 1.0344 strain displayed the highest Pb binding capability (**Table 2**) and showed a significant forbearance against the Pb. Additionally, PRS based microencapsulated *L. plantarum* KLDS 1.0344 presented a remarkable survival in the simulated gastrointestinal conditions (data not shown). Due to the structural differences in bacterial species different metal biosorption mechanisms have been proposed, including micro precipitation and chelation, ion exchange, complexation, and adsorption (Monachese et al., 2012). The Gram-positive bacteria contain polysaccharides, proteins, peptidoglycans, and teichoic acids in their cell walls (Schär-Zammaretti and Ubbink, 2003; Passot et al., 2015). The negatively charged functional groups are present in these contents. These functional groups act as primary sites on a bacterial surface of metal ion sorption (Mishra et al., 2013; Yadav et al., 2014). On cell surface of bacteria the carboxyl and phosphate groups (present in teichoic acids and peptidoglycans) are the key sites to bind metal ions (Kazy et al., 2009).

To imitate prolonged contact, we supplied Pb orally by means of drinking water at the concentration of 100 mg/L, the quantity of which was chosen conferring to the normal human and animal Pb ingestion, animal body weight and concentrations in the contaminated zones, for demonstration of the relevant ecological Pb exposure (Fan et al., 2008). Hereby, we have testified that encapsulated *L. plantarum* 1.0344, not only, demonstrated a better Pb chelating capability and might propose substantial protection to counter Pb noxiousness *in vivo* assisted by chelating action of PRS and lessening the Pb intensities in the blood and tissues (**Figure 3C**), as well as, KLDS 1.0344 prevented the variations in the levels of GSH, GSH-Px, MDA, SOD. We noticed that non-encapsulated and encapsulated *L. plantarum* 1.0344 have supremely significant effect on mechanism of Pb noxiousness. High MDA level accelerates the oxidative stress and the lipid peroxidation process. Ultimately affects the antioxidant defense system, by disturbing the enzyme activity of SOD, GSH-Px, and GSH (Tian et al., 2015) discovered the properties of *L. plantarum* CCFM8661 on reducing Pb concentrations in tissues and blood, improving the enzymatic action of antioxidant protection mechanism and easing the oxidative trauma (Li et al., 2017) revealed that *L. bulgaricus*, not only, decreased the oxidative stress induced by Pb, but also, played a protective role by facilitating the reduction in the concentrations of of lipid peroxide and MDA and escalating the levels of other antioxidants, like, GSH, T-SOD, and GSH-Px in the kidneys and liver. The impairment of energy metabolism and mitochondrial dysfunction is due to the depletion of GSH (Pereira and Oliveira, 2000). It was stated that *lactobacilli* could sequester Pb quickly, and the bio-sorption was steady *in vitro*, and hence was not smooth for desorption (Halttunen et al., 2008; Schut et al., 2011).

Moreover, KLDS 1.0344 by itself did not cause the loss of crucially essential metals, like Mg, Ca, Zn, and Fe from the mice tissues (**Tables 3A,B**). Certain vital components even escalated in concentration, like Fe in the kidneys and Ca in the liver. Our study showed that the PRS-based ˆmicencapsulated KLDS 1.0344 therapy group contains the higher capability to excrete greater quantity of Pb through feces than non-encapsulated KLDS 1.0344 groups. Analogous results were witnessed for the other *lactobacilli*, which could restrain absorption of heavy metals in intestine in mice by excreting heavy metals through feces (Jomova and Valko, 2011; Zhai et al., 2013, 2014). Huang et al. (2011) and Liu et al. (2018) stated that the modified starches retain the capability of sequestering Pb (Pb^2+^) and mercury (Hg^2+^) ions. Normally, the eminent adsorption capabilities of (modified) starches for Hg^2+^ and Pb^2+^ ions are 131.2 and 123.2 mg/g, correspondingly.

Moreover, San Keskin et al. (2018) observed that cyclodextrin fiber encapsulated *Lysinibacillus* sp. has high efficiency to remove the heavy metals(70 ± 0.2%, 58 ± 1.4%, and 82 ± 0.8% for 30 mg L^-1^ concentrated solutions of the Ni(II), Cr(VI), respectively). Concentrations of 30 mgL^-1^ are already above the acceptable limits of environmental concerns. The obtained results suggested that the cyclodextrin CD-F encapsulated *Lysinibacillus* sp. bio-composite system was appropriately suitable for the potential application in Ni (II), Cr (VI), and reactive dye bio-removal.

Numerous research works have described a probable relation between disruption of metal ion homeostasis and the oxidative stress (Valko et al., 2005; Jomova and Valko, 2011; Odewabi and Ekor, 2017). AST/ALT is an imperative biochemical indicator and alterations in the AST and ALT levels are frequently utilized for liver pathological studies (Vijayavel and Balasubramanian, 2006; Agrahari and Gopal, 2009). Liver cell plasma is the main place where ALT exists. Levels of ALT are elevated in the blood by changing the permeability of liver-cell-membrane when the damage of the liver cell is lower. While in contrast, the increasing levels of AST in the blood are due to the severe damage to the liver cells data presented in **Figure 4F**. On the basis of this discussion, it could be concluded that elevated AST/ALT ratios indicate a severe damage to the liver cells. Li et al. (2017), Liu et al. (2018), and San Keskin et al. (2018) also described that the treatment of LAB like, *L. bulgaricus* could possibly improve the levels of ALT and AST, and encapsulation of LAB with modified starches could increase the capability of these bacteria to adsorb the heavy metals by proving them better choice to be adopted as natural bio-sorbents.

## Conclusion

Lead is a lethal heavy metal and contrasting to other noxious compounds this is barely degradable. Consequently, chronic exposure even to very minute quantity could be resulted in developing adversative health effects. Taking into consideration the other effects, such as oxidative stress, body weight gain, histopathological changes and renal, hepatic damage biomarkers, we are certain of that PRS-based microencapsulated KLDS 1.0344 treatment is safe. Additionally, the intestinal pH is about 7–8 slightly alkaline, which increased the capability of PRS and KLDS 1.0344 for detoxication of Pb^++^ *in vivo* study. Both PRS and KLDS 1.0344 contributed to alleviate the Pb levels in mice body by fecal elimination, minimal absorption of Pb to the blood, maintaining the antioxidant defense system, eventually safeguarded the major body organs from Pb toxicity. The restoration of these natural parameters in the mice with the oral administration of the PRS-based microencapsulated KLDS 1.0344 further approves the protecting properties of this probiotic against prolonged and chronic Pb toxicity.

## Author Contributions

ZM has designed and carried out the present research work, conducted experiments, and has written the present manuscript. This is the major part of the thesis research work of ZM. RR helped in analyzing the statistical data, elaborating, and drawing the figures. SZ and HH provided help during the experimentation. AH, AB, YD, and LW also helped with technical issues, provided help in proofreading and presenting the work in its present form. SP provided place, lab facilities, and funding for the present work.

## Conflict of Interest Statement

The authors declare that the research was conducted in the absence of any commercial or financial relationships that could be construed as a potential conflict of interest.

## References

[B1] Abou-Shanab R. A. I. van Berkum P. Angle J. S. (2007). Heavy metal resistance and genotypic analysis of metal resistance genes in gram-positive and gram-negative bacteria present in Ni-rich serpentine soil and in the rhizosphere of *Alyssum murale*. *Chemosphere* 68 360–367. 10.1016/j.chemosphere.2006.12.051 17276484

[B2] Agrahari S. Gopal K. (2009). Fluctuations of certain biochemical constituents and markers enzymes as a consequence of monocrotophos toxicity in the edible freshwater fish, *Channa punctatus*. *Pest. Biochem. Physiol.* 94 5–9. 10.1016/j.pestbp.2009.02.001

[B3] Al-Khfaji I. N. Fakhrildin M. B. Al-Ani I. M. Mangalo H. H. Al-Obaidi S. R. (2011). The effect of lead exposure of mice during pregnancy on the concentration and motility of epididymal and testicular spermatozoa in offspring mature male mice. *Int. Med. J.* 10 37–42.

[B4] Ahyayauch H. Sansar W. Rendón-Ramírez A. Goñi F. M. Bennouna M. Gamrani H. (2013). Effects of chronic and acute lead treatments on the biophysical properties of erythrocyte membranes, and a comparison with model membranes. *FEBS Open Biol.* 3 212–217. 10.1016/j.fob.2013.04.001 23772396 PMC3668517

[B5] Ashraf U. Tang X. (2017). Yield and quality responses, plant metabolism and metal distribution pattern in aromatic rice under lead (Pb) toxicity. *Chemosphere* 176 141–155. 10.1016/j.chemosphere.2017.02.103 28264775

[B6] Bayer G. (2015). Martindale: the complete drug reference. 38th ed. *Aust. Prescr.* 38:59 10.18773/austprescr.2015.023

[B7] Bhakta J. N. Munekage Y. Ohnishi K. Jana B. B. (2012). Isolation and identification of cadmium- and lead-resistant lactic acid bacteria for application as metal removing probiotic. *Int. J. Environ. Sci. Technol.* 9 433–440. 10.1007/s13762-012-0049-3

[B8] Bhat M. A. Chisti H. Shah S. A. (2015). Removal of heavy metal ions from water by cross-linked potato Di-starch phosphate polymer. *Sep. Sci. Technol.* 50 1741–1747. 10.1080/01496395.2014.978469

[B9] Bidlack W. R. (2002). Casarett & Doull’s toxicology: the basic science of poisons, 6th ed. *J. Am. Coll. Nutr.* 21 289–290. 10.1080/07315724.2002.10719223

[B10] Blennow A. Bay-Smidt A. M. Olsen C. E. Møller B. L. (2000). The distribution of covalently bound phosphate in the starch granule in relation to starch crystallinity. *Int. J. Biol. Macromol.* 27 211–218. 10.1016/S0141-8130(00)00121-5 10828367

[B11] Blennow A. Houborg K. Andersson R. Bidzińska E. Dyrek K. Łabanowska M. (2006). Phosphate positioning and availability in the starch granule matrix as studied by EPR. *Biomacromolecules* 7 965–974. 10.1021/bm050919g 16529438

[B12] Bruschi M. L. Cardoso M. L. Lucchesi M. B. Gremião M. P. (2003). Gelatin microparticles containing propolis obtained by spray-drying technique: preparation and characterization. *Int. J. Pharm.* 264 45–55. 10.1016/S0378-5173(03)00386-7 12972335

[B13] Brzóska M. M. Kamiński M. Supernak-Bobko D. Zwierz K. Moniuszko-Jakoniuk J. (2003). Changes in the structure and function of the kidney of rats chronically exposed to cadmium. I. Biochemical and histopathological studies. *Arch. Toxicol.* 77 344–352. 10.1007/s00204-003-0451-1 12799774

[B14] Ciesielski W. Tomasik P. (2004a). Complexes of amylose and amylopectins with multivalent metal salts. *J. Inorg. Biochem.* 98 2039–2051. 10.1016/j.jinorgbio.2004.09.010 15541493

[B15] Ciesielski W. Tomasik P. (2004b). Werner-type metal complexes of potato starch. *Int. J. Food Sci. Technol.* 39 691–698. 10.1111/j.1365-2621.2004.00828.x

[B16] Cieśla K. Sartowska B. Królak E. (2015). SEM studies of the structure of the gels prepared from untreated and radiation modified potato starch. *Radiat. Phys. Chem.* 106 289–302. 10.1016/j.radphyschem.2014.08.011

[B17] de Vos P. Faas M. M. Spasojevic M. Sikkema J. (2010). Encapsulation for preservation of functionality and targeted delivery of bioactive food components. *Int. Dairy J.* 20 292–302. 10.1016/j.idairyj.2009.11.008

[B18] Dobrakowski M. Pawlas N. Hudziec E. Kozłowska A. Mikołajczyk A. Birkner E. (2016). Glutathione, glutathione-related enzymes, and oxidative stress in individuals with subacute occupational exposure to lead. *Environ. Toxicol. Pharmacol.* 45 235–240. 10.1016/j.etap.2016.06.008 27331344

[B19] Dragan E. S. Apopei Loghin D. F. Cocarta A. I. (2014). Efficient sorption of Cu^2+^by composite chelating sorbents based on potato starch- Graft -Polyamidoxime embedded in chitosan beads. *ACS Appl. Mater. Interfaces* 6 16577–16592. 10.1021/am504480q 25191990

[B20] Fan T. Liu Y. Feng B. Zeng G. Yang C. Zhou M. (2008). Biosorption of cadmium(II), zinc(II) and lead(II) by *Penicillium simplicissimum*: Isotherms, kinetics and thermodynamics. *J. Hazard. Mater.* 160 655–661. 10.1016/j.jhazmat.2008.03.038 18455299

[B21] Fewtrell L. J. Prüss-Üstün A. Landrigan P. Ayuso-Mateos J. L. (2004). Estimating the global burden of disease of mild mental retardation and cardiovascular diseases from environmental lead exposure. *Environ. Res.* 94 120–133. 10.1016/S0013-9351(03)00132-4 14757375

[B22] Flora G. Gupta D. Tiwari A. (2012). Toxicity of lead: a review with recent updates. *Interdiscip. Toxicol.* 5 47–58 10.2478/v10102-012-0009-2 23118587 PMC3485653

[B23] Gupta K. Chatterjee C. Gupta B. (2012). Isolation and characterization of heavy metal tolerant Gram-positive bacteria with bioremedial properties from municipal waste rich soil of Kestopur canal (Kolkata), West Bengal, India. *Biologia* 67 827–836. 10.2478/s11756-012-0099-5

[B24] Halttunen T. Collado M. C. El-Nezami H. Meriluoto J. Salminen S. (2008). Combining strains of lactic acid bacteria may reduce their toxin and heavy metal removal efficiency from aqueous solution. *Lett. Appl. Microbiol.* 46 160–165. 10.1111/j.1472-765X.2007.02276.x 18028332

[B25] Halttunen T. Salminen S. Tahvonen R. (2007). Rapid removal of lead and cadmium from water by specific lactic acid bacteria. *Int. J. Food Microbiol.* 114 30–35. 10.1016/j.ijfoodmicro.2006.10.040 17184867

[B26] Hosseini A. Sharifi A. M. Abdollahi M. Najafi R. Baeeri M. Rayegan S. (2015). Cerium and yttrium oxide nanoparticles against lead-induced oxidative stress and apoptosis in rat hippocampus. *Biol. Trace Elem. Res.* 164 80–89. 10.1007/s12011-014-0197-z 25516117

[B27] Huang C. Lai C. Xu P. Zeng G. Huang D. Zhang J. (2017). Lead-induced oxidative stress and antioxidant response provide insight into the tolerance of *Phanerochaete chrysosporium* to lead exposure. *Chemosphere* 187 70–77. 10.1016/j.chemosphere.2017.08.104 28841433

[B28] Huang D.-L. Wang R.-Z. Liu Y.-G. Zeng G.-M. Lai C. Xu P. (2015). Application of molecularly imprinted polymers in wastewater treatment: a review. *Environ. Sci. Pollut. Res.* 22 963–977. 10.1007/s11356-014-3599-8 25280502

[B29] Huang L. Xiao C. Chen B. (2011). A novel starch-based adsorbent for removing toxic Hg(II) and Pb(II) ions from aqueous solution. *J. Hazard. Mater.* 192 832–836. 10.1016/j.jhazmat.2011.05.094 21724326

[B30] Ibrahim F. Halttunen T. Tahvonen R. Salminen S. (2006). Probiotic bacteria as potential detoxification tools: assessing their heavy metal binding isotherms. *Can. J. Microbiol.* 52 877–885. 10.1139/w06-043 17110980

[B31] Javed M. Ahmad I. Usmani N. Ahmad M. (2016). Studies on biomarkers of oxidative stress and associated genotoxicity and histopathology in *Channa punctatus* from heavy metal polluted canal. *Chemosphere* 151 210–219. 10.1002/star.201500155 26943742

[B32] Jiménez-Pranteda M. L. Poncelet D. Náder-Macías M. E. Arcos A. Aguilera M. Monteoliva-Sánchez M. (2012). Stability of lactobacilli encapsulated in various microbial polymers. *J. Biosci. Bioeng.* 113 179–184. 10.1016/j.jbiosc.2011.10.010 22099374

[B33] Jomova K. Valko M. (2011). Advances in metal-induced oxidative stress and human disease. *Toxicology* 283 65–87. 10.1016/j.tox.2011.03.001 21414382

[B34] Kazy S. K. D’Souza S. F. Sar P. (2009). Uranium and thorium sequestration by a *Pseudomonas* sp.: mechanism and chemical characterization. *J. Hazard. Mater.* 163 65–72. 10.1016/j.jhazmat.2008.06.076 18692958

[B35] Khordad E. Fazel A. Bideskan A. E. (2013). The effect of ascorbic acid and garlic administration on lead-induced apoptosis in rat offspring’s eye retina. *Iran. Biomed. J.* 17 206–213. 10.6091/ibj.1229.2013 23999717 PMC3882924

[B36] Kinoshita H. Sohma Y. Ohtake F. Ishida M. Kawai Y. Kitazawa H. (2013). Biosorption of heavy metals by lactic acid bacteria and identification of mercury binding protein. *Res. Microbiol.* 164 701–709. 10.1016/j.resmic.2013.04.004 23603782

[B37] Lai C. Wang M.-M. Zeng G.-M. Liu Y.-G. Huang D.-L. Zhang C. (2016). Synthesis of surface molecular imprinted TiO_2_ /graphene photocatalyst and its highly efficient photocatalytic degradation of target pollutant under visible light irradiation. *Appl. Surf. Sci.* 390 368–376. 10.1016/j.apsusc.2016.08.119

[B38] Li B. Jin D. Yu S. Etareri Evivie S. Muhammad Z. Huo G. (2017). In vitro and in vivo evaluation of *Lactobacillus delbrueckii* subsp. *bulgaricus* KLDS1.0207 for the alleviative effect on lead toxicity. *Nutrients* 9:845. 10.3390/nu9080845 28786945 PMC5579638

[B39] Li M. Buschle-Diller G. (2017). Pectin-blended anionic polysaccharide films for cationic contaminant sorption from water. *Int. J. Biol. Macromol.* 101 481–489. 10.1016/j.ijbiomac.2017.03.091 28322950

[B40] Liang J. Feng C. Zeng G. Gao X. Zhong M. Li X. (2017a). Spatial distribution and source identification of heavy metals in surface soils in a typical coal mine city, Lianyuan, China. *Environ. Pollut.* 225 681–690. 10.1016/j.envpol.2017.03.057 28363446

[B41] Liang J. Li X. Yu Z. Zeng G. Luo Y. Jiang L. (2017b). Amorphous MnO_2_ modified biochar derived from aerobically composted swine manure for adsorption of Pb(II) and Cd(II). *ACS Sustain. Chem. Eng.* 5 5049–5058. 10.1021/acssuschemeng.7b00434

[B42] Liu Q. Li F. Lu H. Li M. Liu J. Zhang S. (2018). Enhanced dispersion stability and heavy metal ion adsorption capability of oxidized starch nanoparticles. *Food Chem.* 242 256–263. 10.1016/j.foodchem.2017.09.071 29037687

[B43] Mahmoud K. F. Amin A. A. Salama M. F. Seliem E. I. (2015). Encapsulation of nano beta-glucan for preservation of functionality and targeted delivery of bioactive food component. *Int. J. ChemTech Res.* 8 587–598. 10.1016/j.idairyj.2009.11.008

[B44] Mishra V. Balomajumder C. Agarwal V. K. (2013). Dynamic, mechanistic, and thermodynamic modeling of Zn(II) ion biosorption onto zinc sequestering bacterium VMSDCM. *Clean Soil Air Water* 41 883–889. 10.1002/clen.201200532

[B45] Monachese M. Burton J. P. Reid G. (2012). Bioremediation and human tolerance to heavy metals through microbial processes: a potential role for probiotics? *Appl. Environ. Microbiol.* 78 6397–6404. 10.1128/AEM.01665-12 22798364 PMC3426676

[B46] Morcillo P. Esteban M. Cuesta A. (2016). Heavy metals produce toxicity, oxidative stress and apoptosis in the marine teleost fish SAF-1 cell line. *Chemosphere* 144 225–233. 10.1016/j.chemosphere.2015.08.020 26363324

[B47] Muhammad Z. Ramzan R. Huo G. C. Tian H. Bian X. (2017). Integration of polysaccharide-thermoprotectant formulations for microencapsulation of *Lactobacillus plantarum*, appraisal of survivability and physico-biochemical properties during storage of spray dried powders. *Food Hydrocoll.* 66 286–295. 10.1016/j.foodhyd.2016.11.040

[B48] Odewabi A. O. Ekor M. (2017). Levels of heavy and essential trace metals and their correlation with antioxidant and health status in individuals occupationally exposed to municipal solid wastes. *Toxicol. Ind. Health* 33 431–442. 10.1177/0748233716669276 27742903

[B49] Office of Environmental Health Hazard Assessment [OEHHA] (2001). *No Significant Risk Levels (NSRLS) for the Proposition 65 Carcinogens Lead and Lead Compounds (Oral).* California, CA: OEHHA, 19.

[B50] Okoro K. I. Igene J. O. Ebabhamiegbebho P. A. Evivie S. E. (2015). Lead (Pb) and Cadmium ( Cd ) levels in fresh and smoke- dried grasscutter (Thryonomys swinderianus Temminck) meat. *Afr. J. Agric. Res.* 10 3116–3122. 10.5897/AJAR2015.9683

[B51] Passot S. Gautier J. Jamme F. Cenard S. Dumas P. Fonseca F. (2015). Understanding the cryotolerance of lactic acid bacteria using combined synchrotron infrared and fluorescence microscopies. *Analyst* 140 5920–5928. 10.1039/C5AN00654F 26212688

[B52] Patra R. C. Swarup D. Dwivedi S. K. (2001). Antioxidant effects of? alpha tocopherol, ascorbic acid and L-methionine on lead induced oxidative stress to the liver, kidney and brain in rats. *Toxicology* 162 81–88. 10.1016/S0300-483X(01)00345-6 11337108

[B53] Peng H. Zhong S. Lin Q. Yao X. Liang Z. Yang M. (2016). Removal of both cationic and anionic contaminants by amphoteric starch. *Carbohydr. Polym.* 138 210–214. 10.1016/j.carbpol.2015.11.064 26794754

[B54] Pereira C. F. Oliveira C. R. (2000). Oxidative glutamate toxicity involves mitochondrial dysfunction and perturbation of intracellular Ca^2+^ homeostasis. *Neurosci. Res.* 37 227–236. 10.1016/S0168-0102(00)00124-3 10940457

[B55] San Keskin N. O. Celebioglu A. Sarioglu O. F. Uyar T. Tekinay T. (2018). Encapsulation of living bacteria in electrospun cyclodextrin ultrathin fibers for bioremediation of heavy metals and reactive dye from wastewater. *Colloids Surf. B Biointerfaces* 161 169–176. 10.1016/j.colsurfb.2017.10.047 29078166

[B56] Sansar W. Ahboucha S. Gamrani H. (2011). Chronic lead intoxication affects glial and neural systems and induces hypoactivity in adult rat. *Acta Histochem.* 113 601–607. 10.1016/j.acthis.2010.06.005 20656334

[B57] Sansar W. Bouyatas M. M. Ahboucha S. Gamrani H. (2012). Effects of chronic lead intoxication on rat serotoninergic system and anxiety behavior. *Acta Histochem.* 114 41–45. 10.1016/j.acthis.2011.02.003 21392819

[B58] Schär-Zammaretti P. Ubbink J. (2003). The cell wall of lactic acid bacteria: surface constituents and macromolecular conformations. *Biophys. J.* 85 4076–4092. 10.1016/S0006-3495(03)74820-6 14645095 PMC1303707

[B59] Schut S. Zauner S. Hampel G. König H. Claus H. (2011). Biosorption of copper by wine-relevant lactobacilli. *Int. J. Food Microbiol.* 145 126–131. 10.1016/j.ijfoodmicro.2010.11.039 21195499

[B60] Sidhu G. P. S. Singh H. P. Batish D. R. Kohli R. K. (2016). Effect of lead on oxidative status, antioxidative response and metal accumulation in *Coronopus didymus*. *Plant Physiol. Biochem.* 105 290–296. 10.1016/j.plaphy.2016.05.019 27214085

[B61] Soto D. Urdaneta J. Pernía K. León O. Muñoz-Bonilla A. Fernández-García M. (2015). Heavy metal (Cd^2+^, Ni^2+^, Pb^2+^ and Ni^2+^) adsorption in aqueous solutions by oxidized starches. *Polym. Adv. Technol.* 26 147–152. 10.1002/pat.3439

[B62] Soto D. Urdaneta J. Pernía K. León O. Muñoz-Bonilla A. Fernandez-García M. (2016). Removal of heavy metal ions in water by starch esters. *Starch Stärke* 68 37–46. 10.1002/star.201500155

[B63] Tian F. Xiao Y. Li X. Zhai Q. Wang G. Zhang Q. (2015). Protective effects of lactobacillus plantarum CCFM8246 against copper toxicity in mice. *PLoS One* 10:e0143318. 10.1371/journal.pone.0143318 26605944 PMC4659595

[B64] Tian F. Zhai Q. Zhao J. Liu X. Wang G. Zhang H. (2012). Lactobacillus plantarum CCFM8661 alleviates lead toxicity in mice. *Biol. Trace Elem. Res.* 150 264–271. 10.1007/s12011-012-9462-1 22684513

[B65] Valko M. Morris H. Cronin M. (2005). Metals, toxicity and oxidative stress. *Curr. Med. Chem.* 12 1161–1208. 10.2174/0929867053764635 15892631

[B66] Valverde M. Fortoul T. I. Díaz-Barriga F. Mejía J. del Castillo E. R. (2002). Genotoxicity induced in CD-1 mice by inhaled lead: differential organ response. *Mutagenesis* 17 55–61. 10.1093/mutage/17.1.55 11752234

[B67] Vijayavel K. Balasubramanian M. P. (2006). Fluctuations of biochemical constituents and marker enzymes as a consequence of naphthalene toxicity in the edible estuarine crab *Scylla serrata*. *Ecotoxicol. Environ. Saf.* 63 141–147. 10.1016/j.ecoenv.2005.02.004 16399165

[B68] Yadav A. Mathur R. Samim M. Lomash V. Kushwaha P. Pathak U. (2014). Nanoencapsulation of DMSA monoester for better therapeutic efficacy of the chelating agent against arsenic toxicity. *Nanomedicine* 9 465–481. 10.2217/nnm.13.17 24910877

[B69] Yi Y. J. Lim J. M. Gu S. Lee W. K. Oh E. Lee S. M. (2017). Potential use of lactic acid bacteria *Leuconostoc mesenteroides* as a probiotic for the removal of Pb(II) toxicity. *J. Microbiol.* 55 296–303. 10.1007/s12275-017-6642-x 28361342

[B70] Zhai Q. Wang G. Zhao J. Liu X. Narbad A. Chen Y. Q. (2014). Protective effects of Lactobacillus plantarum CCFM8610 against chronic cadmium toxicity in mice indicate routes of protection besides intestinal sequestration. *Appl. Environ. Microbiol.* 80 4063–4071. 10.1128/AEM.00762-14 24771031 PMC4054212

[B71] Zhai Q. Wang G. Zhao J. Liu X. Tian F. Zhang H. (2013). Protective effects of lactobacillus plantarum ccfm8610 against acute cadmium toxicity in mice. *Appl. Environ. Microbiol.* 79 1508–1515. 10.1128/AEM.03417-12 23263961 PMC3591948

[B72] Zhai Q. Yin R. Yu L. Wang G. Tian F. Yu R. (2015). Screening of lactic acid bacteria with potential protective effects against cadmium toxicity. *Food Control* 54 23–30. 10.1016/j.foodcont.2015.01.037

[B73] Zhang C. Lai C. Zeng G. Huang D. Tang L. Yang C. (2016). Nanoporous Au-based chronocoulometric aptasensor for amplified detection of Pb2+using DNAzyme modified with Au nanoparticles. *Biosens. Bioelectron.* 81 61–67. 10.1016/j.bios.2016.02.053 26921553

[B74] Zhang S. Zhang X. Chang C. Yuan Z. Wang T. Zhao Y. (2016). Improvement of tolerance to lead by filamentous fungus *Pleurotus ostreatus* HAU-2 and its oxidative responses. *Chemosphere* 150 33–39. 10.1016/j.chemosphere.2016.02.003 26891354

